# Thermal Effect, Diffusion, and Leaching of Health-Promoting Phytochemicals in Commercial Canning Process of Mango (*Mangifera indica* L.) and Pineapple (*Ananas comosus* L.)

**DOI:** 10.3390/foods10010046

**Published:** 2020-12-26

**Authors:** Palitha C. Arampath, Matthijs Dekker

**Affiliations:** 1 Department of Food Science & Technology, Faculty of Agriculture, University of Peradeniya, Peradeniya 20400, Sri Lanka; 2 Food Quality and Design Group, Department of Agrotechnology and Food Sciences, Wageningen University, P.O. Box 17, 6700AA Wageningen, The Netherlands; matthijs.dekker@wur.nl

**Keywords:** β-carotene, antioxidant, degradation, activation energy, flavonoids

## Abstract

The effect of thermal processing on health-promoting phytochemicals was investigated in relation to the canning of mango and pineapple. The cans were retorted at four different temperatures for varying amounts of time. Vitamin C, β-carotene, polyphenols, flavonoid content, and antioxidant capacity in canned fruit pieces and sugar syrup were determined. The diffusion and leaching of phytochemicals were determined through mathematical modelling. Retention of vitamin C in canned pineapple pieces was higher than in canned mango pieces. Thermal treatment had an effect of rapidly reducing leached vitamin C. The activation energy required for the degradation of vitamin C and β-carotene in mango was higher than that required in pineapple. Trolox equivalent antioxidant capacity (TEAC) values at 130 °C (40 min) were 3.1 and 1.9 μmol Trolox/g FW in mango pieces and syrups, respectively, indicating that antioxidant leaching had occurred. The degradation constants of the phytochemicals studied were comparable but were slightly higher in pineapple than in mango. The investigated health-promoting phytochemicals in canned products were present in substantially lower concentrations than in fresh mango and pineapple.

## 1. Introduction

Thermal treatment is an extensively applied food processing and preservation technique that extends the shelf life of fruit products [1]. The primarily focus of food processing is to inactivate pathogenic microorganisms and enzymes. However changes in appearance, composition, nutrition, and sensory properties are occurred inevitably. Temperature in food processing is the most important factor that affects alteration and destruction of natural phytochemicals, thus influence the antioxidant capacity in processed foods [2]. Vitamin C, the marker of nutritional quality of processed fruits and vegetables has contributed lower level for thetotal antioxidant activity at higher temperatures in some fruit matrices [3]. The free and conjugated /bound forms of phytochemicals contribute to the total antioxidant activity of fruits and vegetables [4].

Canned fruit products, especially the tropical fruits are higher in demand in the international trade. Canning as a one of the best processing techniques that preserve the natural sensory attributes as preserved in liquid medium, the canned fruit products are in higher consumer perception compared to the dehydrated products. The research studies reported in canning of fruits in the past few decades were mostly focused on optimization of process conditions, shelf life determination, and microbiological safety. However the attention of nutritional quality and health benefits of the canned fruit products were not reported under commercial scale or laboratory scale. Therefore study of commercially important tropical fruits, mango, and pineapple is timely significant to the food industry, food technologists, nutritionists, and general consumers as well.

Canning of mango and pineapple is a major preservation techniques [5]. Natural fruit juices or mixed juices and sugar syrups with arange of concentrations, 10–22 °Bx are used as the filling media in the canning of fruits or fruit pieces. The syrup is classified on the basis of its cut-out strength, as extra light syrup (>10°Bx), light syrup (>14 °Bx), heavy syrup (>18 °Bx), or extra heavy syrup (>22 °Bx). This liquid medium facilitates heat penetration, inactivation of enzymes, and destruction of pathogenic microorganisms [6,7]. Quality parameters, beneficial phytochemicals, and sensory attributes of canned products are mainly affected by leaching, the formation and degradation of compounds, non-enzymatic browning reactions, and pigment destruction [8,9]. Phytochemicals are considered as bioactive non-nutrient plant compounds with health benefits. These bioactive non-nutrients are available in fruits, grains, vegetables, and other plant foods [10].

Mango is a rich source of macronutrients, micronutrients (vitamins and minerals), and non-nutritive phytochemicals such as phenolic compounds, carotenoids, flavonoids, and other polyphenols [11]. Flavonoids represent 60% of dietary polyphenols in plants and potentially possess human cancer preventive abilities [12,13].

Several authors have reported that a diet rich in polyphenolic compounds is associated with protective effects against some chronic degenerative diseases related to oxidative stress, such as cancer and cardiovascular diseases [14]. Gallic acid (major polyphenol) and hydrolysable tannins (*p*-OH-benzoic acid, *m*-coumaric acid, *p*-coumaric acid, and ferulic acid) are present in mangoes [15].

Pineapple is a good source of several phytochemicals, such as flavonoids; coumaric acid, ellagic acid, ferulic acid, chlorogenic acid, and micronutrients and dietary fiber. These nutritional and non-nutritional constituents vary depending on the cultivar and several pre- and post-harvest management practices [16].

Several varieties of canned pineapple products are available on the market, such as chunks, titbits/pieces, and slices. A clinical study on the consumption of canned pineapple reported reductions in the duration and incidence of infection in school children [17]. Kinetic models of thermal treatment are important to maintain new food process designs and to assure the quality and safety of food production [18]. The degradation of, or damage to, nutrients during thermal treatment can be predicted by determining the kinetic behavior of these compounds. Kinetic studies on several compounds in fruits and juices have been conducted, e.g., in orange juice [19], and raspberry pulp [20]. Improvement of food quality with variable retort temperatures [21], global optimization of process conditions in the batch thermal sterilization of food [22], optimization of the sterilization process of canned food using temperature distribution [6], and heat transfer simulation and retort program adjustment for thermal processing [7] have been reported. Studieson degradation kinetic parameters of anthocyanins and mathematical models and estimation of activation energy (Ea), and z-value during the thermal processing of foods and storage were reviewed [10].

Although studies on the process optimization and thermal processing of canning have been reported, information on the fate of the beneficial bioactive compounds during the canning of mango and pineapple is scant. In this study, processing effects on beneficial phytochemicals, retention of compounds, and the kinetics and activation energy of bioactive compounds were determined based on pilot-scale commercial canning of mango and pineapple.

## 2. Materials and Methods 

### 2.1. Raw Materials and Chemicals 

Mango (Variety Alphonso, Maharashtra, India) and pineapple (Variety Kew, Huetar Norte region, Costa Rica) fruits were purchased from the open market in Wageningen, the Netherlands. Ethanol, methanol, tetrahydroxyfuran (THF), Hydroxymethylfurfural (HMF), acetonitril (BioSolve, Valkenswaard, The Netherlands), trolox, tannic acid, 1,1-diphenyl-2-picrylhydrazyl (DPPH), chlorogenic acid, epicatechin, catechine (Sigma-Aldrich Chemie GmbH, Steinheim, Germany), caffeic acid, sinapic acid and gallic acid (Fluka, Feinchemikalien GmbH, Germany), trifluoroacetic acid (TFA), 2, 6 dichlorophenol-indophenol (DCP), β-apo-8-carotenal, Butylated Hydroxy Toluene (BHT), Folin–Ciocalteu reagent, magnesium carbonate, anhydrous sodium sulphate and triethylamine (Merck, Darmstadt, Germany) were used for the chemical analysis.

### 2.2. Preparation of Fruits for Analysis

Healthy mango and pineapple fruits were naturally ripened at ambient temperature (18 ± 2 °C) for 5 days. Subsequently, they were washed in potable running water, knife-peeled, and cut into cubes of 2.5–3.0 cm. The filling medium, sugar syrup (15 °Bx), was prepared using filtered potable water to avoid physical contaminants. The empty cylindrical metal cans (nominal capacity 454–457 mL) were washed before filling. The empty cans were filled with fruit pieces and sugar syrup, leaving 10% headspace. Uniform weight (net wt. 280 ± 2 g, gross wt. 450 ± 2 g) and filling height were maintained in cans. Filled cans were exhausted in a hot water bath at 80 ± 1 °C for 20 min and immediately sealed (Anico-Junior sealer machine, UK) to create a vacuum in the headspace. The sealed cans were thermally treated using pressurized steam (1.5–3.0 bar) in a pilot scale rotary-type retort (BXF-S511X model, UK). The rotary-type retort is practically desirable in the canning process due to its efficient internal heat transfer due to continuous rotating and mixing action. Temperature–time combinations for the canning experiments were 115,120, 125, and130 °C (retort temperature) and 10, 20, 30, and 40 min thermal treatments. At the end of the thermal treatments, cans were cooled immediately using running water to avoid the over-cooking of fruit pieces. Then, cans were air-dried and stored (4 weeks) at ambient temperature (18 ± 2 °C) for further analysis. This time period is required to stabilize the constituents in solid–liquid matrices in the retorted cans and to detect any defects in cans such as bulged cans. A data logger (iButton, Thermochron type DS1922T, USA) was used to record the internal can temperature at the coldest spot of a filled can under different time–temperature combinations. 

### 2.3. Analysis of Constituents 

Total soluble solids (TSS), vitamin C, total polyphenols, antioxidant activity (Trolox equivalent antioxidant capacity, TEAC), β-carotene, and flavonoids were determined in fresh fruits, canned fruit pieces, and syrup.

#### 2.3.1. Determination of Total Soluble Solids (TSS)

The TSS (°Bx) value was measured with a hand-held refractometer (Eclipse, code 45-91, UK).

#### 2.3.2. Determination of Vitamin C Content

The vitamin C (L-ascorbic acid) content was determined using the Colorimetric method with an Enzytec™test kit, ID No: 1 002 941.

#### 2.3.3. Determination of Total Polyphenolic Content

The total polyphenolic content was determined using Folin–Ciocalteu’s reagent [23]. A calibration curve was constructed using gallic acid. The determined quantity is presented in mg gallic acid equivalents per 100 g in fresh weight (mg GAE/100 g FW). 

#### 2.3.4. Antioxidant Activity 

The free radical scavenging capacity of the different antioxidants in the samples was measured using a DPPH (1,1-diphenyl-2-picrylhydrazyl) assay, as explained by [24] and later modified for measuring lipophilic compounds [25]. The antioxidant capacity was expressed as the Trolox Equivalent Antioxidant Capacity (TEAC) in micro moles per gram of fresh weight (μmol/g FW).

#### 2.3.5. Determination of β-Carotene Content 

β-Carotene content was determined by Reversed-Phase High-Performance Liquid Chromatography (RP-HPLC) technique. The extraction method of β-carotene was a slightly modified version of the method [26,27].

#### 2.3.6. Determination of Flavonoid Content

Flavonoid compounds were quantified using HPLC analysis [28]. Standard phenolic compounds, tannic acid, chlorogenic acid, caffeic acid, epicatechin, catechin, gallic acid, sinapic acid, and a non-phenolic compound, Hydroxymethylfurfural (HMF) were used. Quantification was performed using the calibration curves, while identification was achieved by comparison of the peaks in the standard solutions with the samples.

#### 2.3.7. Mathematical Modelling

The concentrationof biochemical components in the cellular matrix of fruit can be affected by several mechanisms during canning. Cell lysis in fruit pieces, leaching of components into the liquid phase of the cans, diffusion upon heating, and thermal degradation are the possible mechanisms in the liquid phase at elevated temperatures during the canning process.

The mathematical model used for the canning process only includes the effects of lysis/leaching and thermal degradation in the fruit matrix and in the can filling medium (sugar syrup 15 °Bx). Cell lysis is described by a first-order kinetic model (Equation (1)), as this was also observed for the lysis of vegetable cells [29]. A mass balance was used to relate the fraction of lysed cells to the fraction of intact cells (Equation (2)):

(1) $$\frac{dC_{c,i}}{dt}=-k_{l}.C_{c,i}.\;$$

(2) $$C_{c,l}=1-C_{ci}$$

where

*C_c,i_*:  fraction of intact cells (-),
*C_c,l_*:  fraction of lysed cells (-),
*k_l_*:  lysis rate constant (min^−1^),
*t*:  time (min).

For the leaching of water-soluble components to occur, cells must lyse and the components must diffuse to the surrounding water. Due to the heterogeneous size and shape of the fruit particles, the diffusion process is difficult to model exactly. For practical reasons, it is therefore assumed that the lysis and diffusion processes can be described together by first-order equations and mass balance (Equations (1) and (2)). The lysis rate constant, in fact, is a lumped parameter also including the diffusion process. The resulting parameter (*k_l_*) is therefore called the leaching parameter. By making this assumption, the transfer of components to the surrounding water can be described by a mass balance of the amount of water in the fruit (in intact cells and lysed cells) and the surrounding water.The volume of the free water phase (surrounding water plus water in lysed cells), in fact, increases as more cell are lysed. This process can be described by the following relation (Equation (3)):

(3) $$\frac{dM_{w}}{d_{t}}=-\frac{dC_{c,i}}{d_{t}}.M_{f,0}=\;k_{l}.C_{c,i}.{\;M}_{f,o}$$

where

*M_w_*: mass of free water (g),
*M_f,_*_0_: initial mass of fruit (g).

According to this model, the leaching of water-soluble components is a direct consequence of cell lysis. The contents of the cells that lyse are added to the free water phase. To describe this process mathematically, one has to take into account the amount of components from the lysed cells as well as the diluting effect caused by the increase in the mass of free water caused by this lysis (Equation (4)):

(4) $$\frac{dC_{w}}{d_{t}}I_{L}=\;\left[\frac{k_{l}\;.{\;C}_{c,i}\;.{\;M}_{f,0}\;.C_{v}}{M_{w}}\right]-\left[C_{w}.\frac{k_{l}.{\;C}_{c,i}\;.{\;M}_{f,0}}{M_{w}}\right]=\left[\frac{k_{l}\;.{\;C}_{c,i\;}.{\;M}_{f,0\;}.\;(C_{f-\;C_{w})}}{M_{w}}\right]$$

where

*_L_*: refers to the fact that this component represents the change due to leaching only,
*C_w_*: component concentration in the free water (µmol/g),
*C_f_*: component concentration in the intact part of the fruit (µmol/g).

Thermal breakdown is described by first-order kinetics, similar to previous studies [30]. Since breakdown rates can be different in fruit and the surrounding water, different rate constants can be used (Equations (5) and (6)):

(5) $$\frac{dC_{f}}{d_{t}}=-k_{d,f}.C_{f}$$

(6) $$\frac{dC_{w}}{d_{t}}I_{B}=-k_{d,w}.C_{w}$$

where

*_B_*: refers to the fact that this component is the change due to breakdown only,
*k_d__,f_*: breakdown rate constant in fruit (min^−1^),
*k_d__,w_*: breakdown rate constant in water (min^−1^).

All estimations of the reaction rates were performed on the respective, experimentally determined individual concentrations of each canning experiment and not by using the average values of the three consecutive runs, effectively accounting for sample variability. The rate constants for leaching and degradation are temperature-dependent. This fact is described by a modified Arrhenius equation, which determines the rate constant relative to a reference temperature (Equation (7)):

(7) $$k_{d}=k_{d,ref}\;e^{\left(\frac{E_{a}}{R}\right)\left(\frac{1}{T_{ref}\;}-\;\frac{1}{T}\right)}$$

All equations were fitted to data of individual concentrations in both water and fruit pieces simultaneously using the Athena Visual Workbench software (Athena Visual Studio, Inc, Naperville, IL, USA). Reaction kinetics were studied by multi-response modelling using the determinant criterion [31]. Multi-response modelling implies that more than one reactant or product is taken into account. The determinant criterion is then more suitable than the familiar least-squares criterion. Athena Visual Workbench was used for numerical integration of differential equations, as well as parameter estimation of the rate constants in the differential equations, following minimization of the determinant in order to obtain the reaction kinetic parameters (rate constant *k_a_* and activation energy *E*_a_). The goodness of fit of the models, describing first-order degradation, was calculated using the Pearson chi-square test.

## 3. Results and Discussion

**Figure 1:**
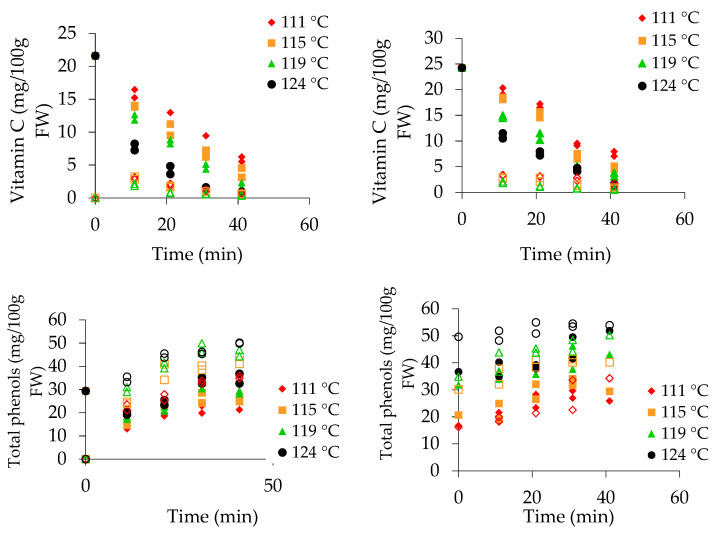

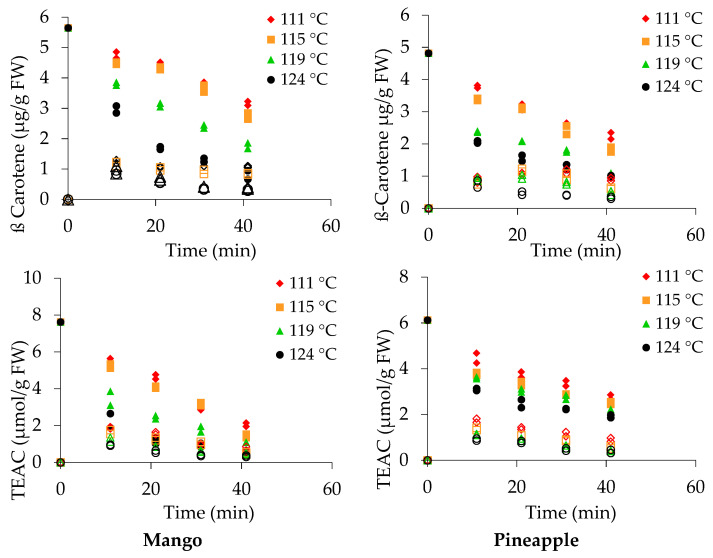
Vitamin C, total phenols, β-carotene, and trolox equivalent antioxidant capacity (TEAC) in pieces (closed symbols) and syrup (open symbols) during the canning of mango and pineapple at different temperatures. The legend represents the steady state coldest spot temperature in the can measured by the iButton.

The vitamin C, total polyphenol, and β-carotene contents and the TEAC in mango and pineapple pieces and sugar syrup measured underdifferent time–temperature combinations are given in Figure 1. The legend represents the steady-state temperature (internal can temperature) at the coldest point of a can, measured during thermal treatment in the pilot scale retort. The recorded internal can temperatures of 111, 115, 119, and 124 °C corresponded to the retort temperatures of 115, 120, 125, and 130 °C. 

### 3.1. Vitamin C Content

The vitamin C content in ripe fresh mangoes used for canning was 21.6 ± 1.2 mg/100 g. The value is within the previously reported range (19.7 ± 9.1–38.7 ± 1.4 mg/100 g) [32,33]. The vitamin C content in pineapple (24.3 ± 1.1 mg /100 g) was similar to that reported previously [34,35]. The vitamin C content was reduced in canned mango and pineapple pieces during thermal treatment (Figure 1). At the end of the heat treatment at 115 °C for 40 min, the retention of vitamin C was 27% in mango pieces and 31% in pineapple pieces. The retention of vitamin C in mango pieces ranged from 4% (130 °C) to 27% (115°C) after 40 min of thermal treatment. In canned pineapple, the retention rate was 6% at 130 °C and 31% at 115 °C under the same conditions. Loss of vitamin C occurs by non-enzymatic reactions, oxidation during fruit peeling, cutting, and can exhausting. Further, leaching of vitamin C into sugar syrup and rapid reduction was evident. Ascorbic acid shows a high reduction property, and the reaction rate is strongly affected by the dissolved oxygen concentration. Differentiation of the ascorbic acid (AA) decomposition mechanism under two conditions, in the presence and absence of oxygen, was established [36]. Variation in kinetic parameters resulted from the intrinsic characteristics of the product such as its maturity, variety, pH, and dissolved oxygen level. A reduction in the concentration of dissolved oxygen in citrus juice after thermal treatment was reported [37].Therefore, a slower degradation reaction rate would be expected even at higher temperatures under anaerobic conditions inside the cans during the can exhausting step. This was proven by the estimated *E*_a_ values: 109 ± 6 kJ mol^−1^ (mango) and 81 ± 6 kJ mol^−1^ (pineapple). The *E*_a_ values were higher than the reported values (*E*_a_ 36–71 kJ mol^−1^) for some other fruits [19,37]. The rate constant (*k*_a_ = 2.4 ± 0.1 × 10^−2^ min^−1^) was same for both mango and pineapple fruit pieces.

The vitamin C content measured in the sugar syrup of canned mango was 2.7 (115 °C), 3 (120 °C), and 2 mg/100 g (125 °C), and it was undetectable at 130 °C after 10 min of thermal treatment. In sugar syrup of pineapple cans, concentrations of 2.8 mg/100 g (115 °C), 1.6 mg/100 g (120 °C), and 1.2 mg/100 g (125 °C) were measured after 10 min, and vitamin C was undetectable at 130 °C.

### 3.2. Polyphenol Content

The total polyphenol content in mango, 29.4 mg GAE/100 g FW, was lower than previously reported values reported [35,38]. The polyphenol content of 33.3 mg GAE/100 g FW in pineapple is comparable with a previously reported value [34]. In our experiments, an increment in the total polyphenol content was found in mango and pineapple pieces and in sugar syrup with increasing thermal treatment (Figure 1).

The total polyphenol content in fresh mango (29.4 mg/100 g FW) was slightly reduced to 22.8 mg/100 g at 115 °C and gradually increased with an increasing temperature: 25.7 at 120 °C, 28.8 at 125 °C, and 34.7 mg/100 g FW at 130 °C after 40 min of thermal treatment. The retention percentages of polyphenols were 77%, 87%, 98%, and 118%, respectively, with the same temperature and time combinations. Similar behaviour was observed for canned pineapple. The retention percentages in pineapple pieces were 79%, 92%, 134, and 152%, respectively, with the same thermal treatments. The above increments at 119 and 124 °C could be due to the release of hydrolysable polyphenols from the cellular matrices at high temperatures. In fruits, hydrolysable tannins are present as either soluble-free or bound forms as gallo-tannins and ellagitannins [10]. The total phenolic content in pineapple (94.3 ± 1.5 mg/100 g) includes 42.9% soluble-free and 57.1% bound forms [39]. In our canning experiments, the total polyphenol content increased due to transformation of bound phenolics into soluble, detectable forms with potential antioxidant capacity. The hydrolysable compounds in mango and pineapple are responsible for increasing the total polyphenol content during the process of canning fruit pieces. Obviously, the total polyphenol content in the sugar syrup of canned mango and pineapple was higher than in pieces (Figure 1). The reason for this is due to the naturally present or later formed hydrolysable polyphenols being leached from pieces to the syrup with the progression of heat treatment.

### 3.3. β-Carotene

The β-carotene content in fresh mango (5.65 μg/g FW) was within the range 1.7–18 µg/g reported by [40]. The β-carotene content determined in fresh pineapple (4.82µg/g FW) was higher than that reported [28,41]. The retention percentage of β-carotene in mango and pineapple pieces was 56–14% and 47–20%, respectively at 115–130 °C following retorting for 40 min. Further, the degradation of β-carotene increased with the duration of thermal treatment. Leaching of β-carotene from pieces to sugar syrup occurs during canning (Figure 1). β-Carotene contents in sugar syrup of 1.25 μg/g FW and 0.91 μg/g FW were measured at 115 °C after 10 min of retorting in canned mango and pineapple, respectively. A loss of β-carotene, 17% in canned mango was reported [42], and the value was comparable with our finding at 130 °C.

### 3.4. Antioxidant Activity (TEAC)

A range of bioactive compounds; carotenoids, polyphenols, tocopherols, flavonoids, and micro elements contributes to the total antioxidant capacity represented by the synergistic effect of diverse bioactive compounds [43].

The TEAC in fresh mango and pineapple was 6.1 ± 0.3 and 7.6 ± 0.2 μmol Trolox/g FW, respectively. The gradual reduction in TEAC in pieces and syrup could be due to the thermal effects (Figure 1). TEAC values in mango pieces and syrup at 115 °C (10 min.) and 130 °C (40 min) were 4.6 and 2.7 μmol Trolox/g FW and 3.1 and 1.9 μmol Trolox/g FW, respectively.

The measured TEAC values ranged from 0.9 to 1.8 μmol Trolox/g FW in sugar syrup of canned mango at 115–130 °C after 10 min. These values represent the antioxidant activity of leached compounds from the pieces into sugar syrup. The TEAC level in sugar syrup was less than 1 μmol Trolox/g FW after 40 min for all temperature treatments for both fruit types. However, the retention of TEAC in mango pieces was higher than that in pineapple pieces. Pineapple pieces have a softer and more succulent texture, and therefore leaching of constituents from the cellular matrix is more frequent than in mango pieces. Retention of TEAC in mango and pineapple pieces was 45–31% and 27–7.6% following thermal treatment at 115–130 °C for 40 min.

### 3.5. Flavonoids

#### 3.5.1. Mango Fruit Pieces

**Figure 2:**
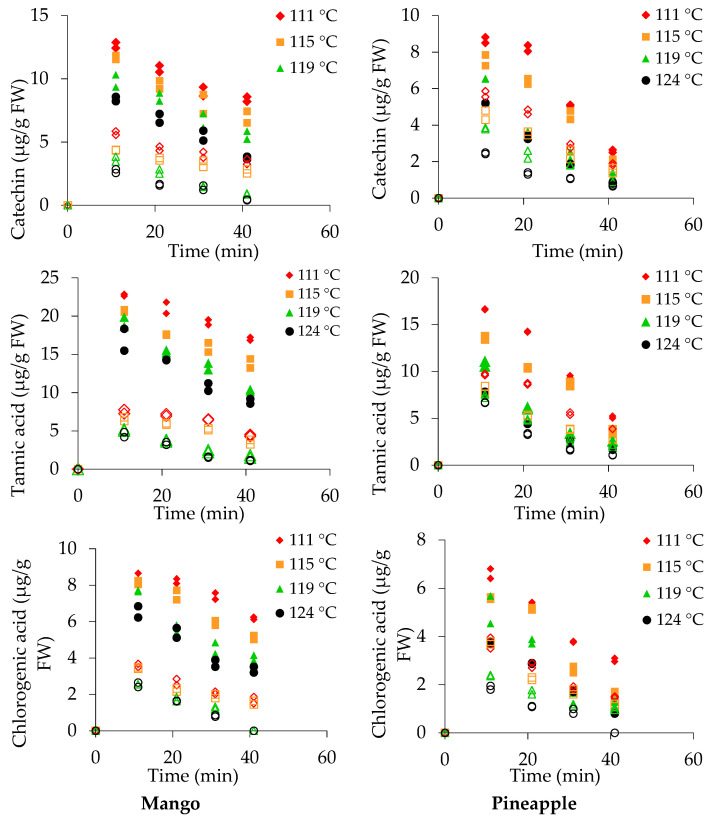
Catechin, tannic acid, and chlorogenic acid concentrations in mango and pineapple pieces (closed symbols) and syrup (open symbols) during canningat different temperatures.

**Figure 3:**
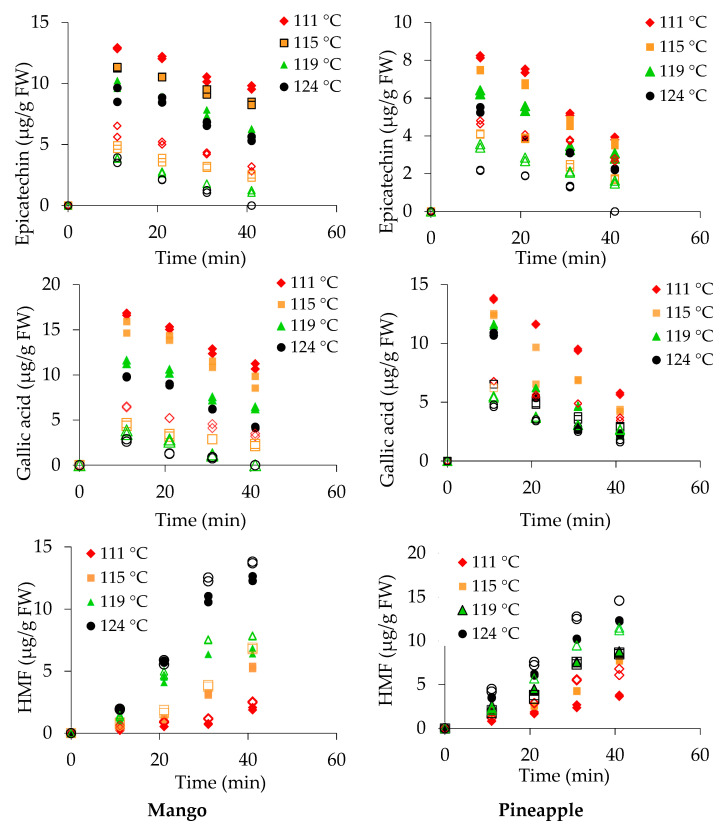
Epicatechin, gallic acid, and Hydroxymethylfurfural (HMF) concentrations in mango and pineapple pieces (closed symbols) and syrup (open symbols) during canningat different temperatures.

Flavonoids measured in canned fruit pieces and sugar syrup at different thermal treatments are shown in Figure 2 and Figure 3. In fresh mango, catechin (42.36 μg/g), tannic acid (48.32 μg/g), chlorogenic acid (18.14 μg/g), epicatechin (26.35 μg/g), and gallic acid (24.53 μg/g) were measured in FW. The content of gallic acid in mango was higher than the previously reported value of 6.9 μg/g [44]. The concentrations of these compounds reduced substantially during fruit preparation for canning and can exhausting followed by retorting. Therefore, the values were 50% lower than the values in fresh fruits after 10 min. A retention rate of catechin, 9–20% was observed in both mango and pineapple pieces at 115 and 130 °C.

Retention of tannic acid in canned mango pieces was greater than retention of catechin (Figure 2). The retention rate of chlorogenic acid was 19–34% in canned mango pieces following treatment at 115 and 130 °C for 40 min. However, the retention rate of epicatechin was greater than that of the other flavonoid compounds17–44% (epicatechin) compared with 11–27% (gallic acid) at 115–130 °C for 40 min in both canned mango and pineapple.

A non-flavonoid compound 5-hydroxymethylfurfural (HMF) was detected in canned fruit pieces and syrup. HMF was formed due to the thermal effect with available sugars, and the quantity was increased with an increasing retort temperature and time. In this experiment, the HMF levelincreased by sixfold from 115 (10 min) to 130 °C (40 min) in mango pieces (Figure 3).

#### 3.5.2. Pineapple Pieces

The concentrations of catechin (45.9 μg/g), tannic acid (34.5 μg/g), chlorogenic acid (20.5 μg/g), epicatechin (18.4 μg/g), and gallic acid (21.3 μg/g) FW were determined in ripe, fresh pineapple (Figure 2 and Figure 3). The results revealed that the loss of catechinwasgreater in pineapple pieces than in mango pieces. The percentage retention of catechin was 2–6% under all treatments.

A 5–15% retention rate was measured for tannic acid in canned pineapple pieces after retorting at 115–130 °C for 40 min. The chlorogenic acid content was measured as 3 and 0.8 μg/g FW in pineapple pieces after 40 minof retorting at 115 and 130 °C, respectively. Degradation of epicatechin in canned pineapple was greater than in mango pieces. In fresh pineapple, the epicatechin content was 18.4 μg/g FW, and after the thermal treatment, the value reduced to 3.9 and 2.2 μg/g FW at 115 °C and 130 °C, respectively. Total retention of gallic acid was 11–27% in pineapple pieces after retorting at 115–130 °C for 40 min.

The HMF was measured in both fruit pieces and sugar syrup. HMF levels of 3.7 and 12.2 μg/g FW were measured in canned pineapple pieces after thermal treatment at 115 °C (40 min) and 130 °C (40 min), respectively.

#### 3.5.3. Sugar Syrup in Cans

Thermal treatment influences the leaching of flavonoid compounds into sugar syrup. In the sugar syrup of mango cans, concentrations of catechin (5.7 ± 0.2 μg/g), tannic acid (7.5 ± 0.4 μg/g), chlorogenic acid (3.6 ± 0.1 μg/g), epicatechin (6.1 ± 0.6 μg/g), gallic acid (6.4 ± 0.1 μg/g), and HMF (2.5 ± 0.1 μg/g) were measured after retorting at 115 °C (10 min). Catechin and tannic acid contents were greater at 115 °C (10 min), and the contents gradually reduced with increased retorting at 130 °C (40 min), as shown in Figure 2. Chlorogenic acid was not detected after retorting at 125 and 130 °C in the sugar syrup of mango cans, while epicatechin was not detected after retorting at 130 °C. Gallic acid levels of 3.65 ± 0.3 and 2.7 ± 0.2 μg/g FW were only measured at 125 and 130 °C (10 min), respectively. The HMF level was increased fivefold at 115 °C and sevenfoldat 130 °Cwhen the duration was increased from 10 to 40 min.

In the sugar syrup of pineapple cans, the concentrations of catechin, tannic acid, chlorogenic acid, epicatechin, gallic acid, and HMF were determined during retorting at 115 °C (40 min). Except for HMF, all compounds degraded as the retorting temperature and time increased (Figure 2 and Figure 3). Chlorogenic acid and epicatechin were not detected in processed pineapple cans treated at 130 °C for 40 min. The lowest recorded concentration of tannic acid, 1.1 μg/g FW, occurred at 130 °C (40 min), while the lowest gallic acid content (1.7 μg/g) was measured in the syrup of pineapple cans at 130 °C (40 min).

In this experiment, the degradation of leached compounds from the fruit pieces fitted well with the combined model. All of these compounds, except HMF, showed substantial degradation with increasing temperature and time. However, the HMF concentration increasedrapidly from 2.5 (10 min) to 14.6 μg/g (40 min) in the sugar syrup of pineapple cans at 130 °C after 40 min of retorting. Vitamin C is a sugar acid and, therefore, at higher temperatures, it functions to reduce sugars in the Maillard reaction, yielding products that are able to react with amino acids, peptides, and lipids. Furfural, the principal product, participates in aldol reactions with other carbonyl compounds. The accumulation of the degradation product, HMF, is highly dependent on temperature [45].

### 3.6. Kinetics of Compounds

**Figure 4:**
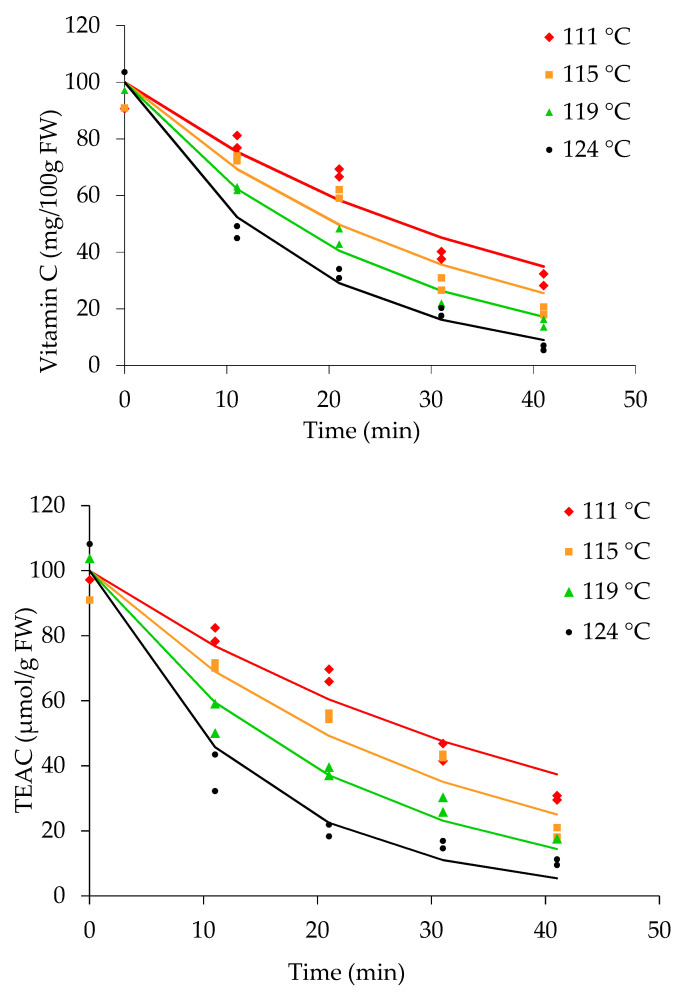
Percentages of vitamin C and TEAC in pineapple pieces expressed as percentages of the original content in initial fruit during canning at different temperatures. Lines are fitted to a model describing first-order degradation using Equations (1) and (2).

The vitamin C content and TEAC in the fruit pieces and sugar syrup, expressed as percentages of the original concentrations in the initial fruits during thermal treatment at 111, 115, 119, and 124 °C (internal can temperatures) are shown in Figure 4. The data were fitted using a first-order degradation model in combination with the modified Arrhenius equation described previously. The goodness of fit of test statistic ($X^{2})$ of the models (Table 1) shows that all derived models fit well with the data (*p* > 0.05), except for TEAC at 124 °C (*p* < 0.05). The rapid degradation of antioxidant compounds at 124 °C at 30–40 min could have more substantially influenced the pattern of degradation than the effects of the other compounds. Further, the kinetic parameters, *k*_a_ and *E*_a_ of TEAC were influenced by the different experimental process conditions. The degradation kinetic parameters *E*_a_ and *k*_d_ calculated at the reference temperature, 111 °C (384 °K), are given in Table 2. The degradation kinetic values for vitamin C (*E*_a_ 81 ± 6 kJ mol^−1^, *k*_d_ 2.4 ± 0.1 × 10^−2^ min^−1^) and TEAC (*E*_a_ 106 ± 8 kJ mol^−1^, *k*_d_ 2.2 ± 0.1 × 10^−2^ min^−1^) were calculated.

The vitamin C content in pineapple pieces decreased as the temperature and retorting time increased. The highest degradation rate was evident at an internal can temperature of 124 °C. A similar finding for the thermal degradation of vitamin C in squeezedoranges heated at 120–150 °C was reported [46]. However, a simple first-order degradation model does not always properly describe the degradation kinetics of vitamin C. In our experiments, the thermal effect on the degradation kinetics of the compounds has been described by a model combining the first-order kinetic model and mass balance applicable for cell lysis/leaching and degradation during the canning of fruit pieces (Equations (1)–(7)).

**Figure 5:**
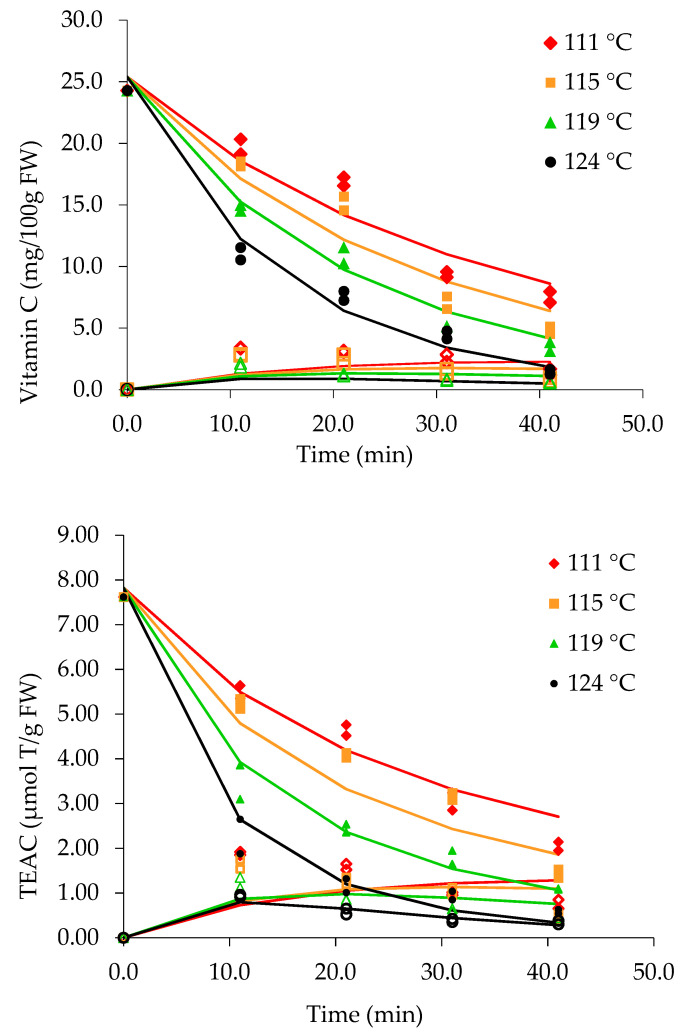
Vitamin C and TEAC concentrations in pineapple pieces (closed symbols) and syrup (open symbols) during canningat different temperatures. Lines are fitted to a model describing degradation and leaching using Equations (1) and (2).

Vitamin C and TEAC concentrations in pineapple pieces and syrup during canning are given in Figure 5. The lines were fitted to a model describing cell lysis, leaching, and degradation by Equations (1)–(7). Degradation can occur in fruit pieces and sugar syrup, as described by the model.Degradation and leaching of vitamin C in canned pineapple pieces gradually increased as the internal can temperature increased. The lowest vitamin C content (1.45 mg/100 g) in pineapple pieces was measured at 124 °C after 40 min. In contrast, the vitamin C content in the sugar syrup of pineapple cans gradually increased due to cell lysis occurring in the pieces followed by leaching to the surrounding liquid matrix.

Leached vitamin C in the sugar syrup is subjected to further degradation during thermal treatment at 111, 115, 119, and 124 °C. The data recorded for sugar syrup fitted the model describing cell lysis, leaching, and degradation (Figure 5). Comparatively, a higher content of vitamin C was measured in the syrup at 111 °C after 10 min, while the lowest content was measured at 124 °C after 40 min. A similar trend was shown in the fitted data for TEAC measured in the sugar syrup.

In our experiments, it is evident that the application of thermal treatment at the lowest temperature preserves a comparatively high content of beneficial health compounds in both fruit pieces and syrup. Therefore, selection of the optimal minimumtime–temperature combination considering microbiological safety is one of the best strategies to ensure the maximal occurrence of healthpromoting compounds in canned fruit products.

The antioxidant activity, as measured by TEAC, in the pieces and syrup is affected by leaching and degradation of compounds. The occurrence of vitamin C and TEAC in fruit pieces and syrup showed similar trends with slight changes. Greater degradation of TEAC in pineapple pieces occurred at 124 °C than at 111–119 °C. The TEAC in sugar syrupshowed a similar trendafter the first 10 min, while more prominent reduction after 10 min was evident at 124 °C than at 111 and 115 °C. The TEAC in pineapple pieces and syrup was stable at 111 °C.β-Carotene, catechin, tannic acid, chlorogenic acid, epicatechin, and gallic acid values measured in both the fruit pieces and sugar syrup are shown in Figure 1, Figure 2 and Figure 3. The degradation kinetics of these compounds were almost the same as those described and shown for vitamin C and TEAC (Figure 5).

The total polyphenol content was comparatively stable. It increased during thermal treatment, as elaborated previously. There is a lack of recent literature on the reaction kinetics of canned mango and pineapple. However, color degradation kinetics during thermal processing of mango and pineapple puree have been reported [47,48].

The degradation rate constant (*k*_a_) and activation energy (*E*_a_) of vitamin C, TEAC, β-carotene, and flavonoids are shown in Table 2. The thermo stability is described by both *E*_a_ and *k*_a_ values. A reaction with a higher *E*_a_ indicates a strong dependency of the rate constant on temperature. Compounds with higher *E*_a_ and lower *k*_a_ values and vice versa show thermo stability. In exceptional situations, higher *E*a and *k*_a_ values in an unstable compound are also associated with thermo stability. In mango pieces, the estimated *E*_a_ value for vitamin C, 109 ± 6 kJ mol^−1^, was higher than that in pineapple pieces (*E*_a_ 81 ± 6 kJ mol^−1^), while *k*_a_ (2.4 ± 0.1 × 10^−2^ min^−1^) was similar in both fruits. In mango pieces, the estimated *E*_a_ value for β-carotene (148 ± 4 kJ mol^−1^) was higher than that in pineapple pieces (92 ± 5 kJ mol^−1^), while *k*_a_ value in mango (0.9 ± 0.0 × 10^−2^ min^−1^) was lower than that in pineapple (1.3 ± 0.1 × 10^−2^ min^−1^). Therefore, the thermo stability of β-carotene is higher in canned mango pieces, and 56% retention in mango was found to be higher than that in pineapple (46%) after thermal treatment at 115°C for 10 min. Similarly, the higher values of *E*_a_ for gallic acid (73 ± 3 kJ mol^−1^) and TEAC (106 ± 8 kJ mol^−1^) in pineapple pieces indicate lower dependency of the degradation rate constants for canned mango pieces.

*E*_a_ values vary based on the type of fruit [20,49]). Variation in the *E*_a_ value (35.9–94.0 kJ mol^−1^) for ascorbic acid degradation has been reported [37]. The *E*_a_ values, determined for vitamin C in this study, 81 ± 6 kJ mol^−1^ (mango) and 109 ± 6 kJ mol^−1^ (pineapple) are within the range previously reported. Leaching or migration of compounds is mainly due to thermal effects on the cell walls and membranes and the exposure of the cut surfaces of fruit to sugar syrup with a low soluble solid content (15 °Bx).The leaching and degradation of vitamin C in pineapple piecesgradually increased as the temperature increased.

In pineapple, the degradation rate constant at 111 °C (*k*_d111_) values of catechin, tannic acid, chlorogenic acid, and epicatechin were higher than those in mango, while the *E*_a_ values were lower than those in mango (Table 2). Therefore, canned mango pieces retain greater amounts of the above tested flavonoids compared with canned pineapple pieces. In canned mango pieces, retention rates of 27% (vitamin C), 78% (polyphenols), 45% (TEAC), and 56% (β-carotene) were determined at 111 °C (40 min), whereas in canned pineapple pieces, there were retention rates of only 31%, 79%, 26%, and 46%, respectively, for the same compounds.

According to the results it is obvious that health benefits of phytochemicalsare generated as additive and/or synergistic effects the complex mixtures of phytochemicals in the fruit matrices rather than a single antioxidant in foods. Measurement of any individual antioxidative compound (such as vitamin C) is potentially biased to determine the antioxidant activity in raw or processed foods. In apples, vitamin C provides only 0.4% of the total antioxidant activity whereas a significant contribution to the total antioxidant activity is contributed by other phytochemicals namely phenolics, flavonoids, and anthocyanins [50]. The results of canned mango and pineapple were comparable with this statement.

The antioxidant activity of canned products has been reduced due to detrimental effects on phenolics and flavonoid compounds at higher thermal treatments. In this study phenolics content was increased with thermal processing as reported by several studies. Therefore further research on the intrinsic properties of the food matrix in relation to the antioxidant activity and thermal effect has to be investigated. Special attention should be given to protect or regain the beneficial phytochemicals those subjected to destruction or loss during peeing and pre-preparatory steps in canning of fruits.

The understanding of mechanism of degradation of phenolic antioxidants during fruit processing at the molecular level, degradation kinetics and characterization should be investigated for designing the process parameters and operations. Further research focusing on retention ofphytochemicals in the processed foods by optimization of thermal and nonthermal processes has potential tooptimize phytochemicals and health benefits. However these studies should be focused to address the preservation of product, food safety and nutritional quality to satisfy the consumers’ perceptions and sustainablemarket demand for thermal processed fruit products in future.

Different processing techniques use diverse mediums of heating. However, influence of available water/liquid in the processed food products has not been investigated adequately during the past. The total antioxidant activity and nutrient contents could be underestimated based on the available quantity of water present in processed foods as elaborated in our study on canned mango and pineapple. In processed foods, the liquid portion is neglected intentionally or unintentionally during analysis.

Authors recommend thermal processing of mango and pineapple using pre-treatments such as steam blanching and/or microwave heating combined with thermal treatment. Higher radical scavenging activity under limited water conditions in microwave cooking of peppers was reported [51]. Retorting of fruit pieces in cans or retortable pouches can be performed with minimum water or syrup content that is sufficient for heat penetration during thermal processing. Thermal effect on product can be potentially reduced and higher retention of bioactive phytochemicals and antioxidant activity can be anticipated.

## 4. Conclusions

The retention of health-promoting compounds was found to be higherin canned mango than in canned pineapple based on the kinetic parameters (*k*_a_ and *E*_a_) determined. Higher temperature treatments may facilitate the cell lysis and leaching of health-promoting compounds into sugar syrup. The estimated *E*_a_ values of vitamin C were 109 ± 6 (mango) and 81 ± 6 kJ mol^−1^ (pineapple) and the *k*_a_ value, 2.4 ± 0.1 × 10^−2^ min^−1^, was the same for both fruits. The total phenolic content in mango increased from 77% (115 °C) to 118% (130 °C) after 40 min, while in pineapple, the content was 79% and 152%. The greater retention at 130°C is due to release of soluble free and hydrolysable bound phenolic compounds in the cellular matrices of both fruit pieces during the expended thermal treatment. There were evidences of leaching and degradation of β-carotene and flavonoids. Accumulation of HMF was highly temperature dependent, and 13.7 and 14.6 µg/g FW in the sugar syrup of both fruit cans processed at 130 °C (40 min) were measured. The first-order degradation model in combination with the modified Arrhenius equation fitted the experimental data very well, as reflected by the goodness of fit test statistic ($X^{2})$ for all variables with the exception of TEAC at 124 °C. The estimated parameters for the degradation kinetics clearly show the differences in behavior of the compounds in different fruits. Processing of mango and pineapple at the lowest optimal temperature with the minimum liquid content in the cans, without disturbing the heat transfer would be applicable for better retention of health promoting phytochemicals. Further, in order to minimize higher thermal treatment and prolong thermal process time, pretreatments such as steam blanching of fruit pieces and microwave treatment can be applied to partial or complete destruction of pathogenic microorganisms and inactivation of enzyme activities in fruit pieces. These pretreated fruit pieces and sugar syrup or fruit juice (heated and cooled previously) are filled in to the sterile cans, followed by conventional canning process. Similarly fruit pieces can be retorted using retort pouches filled with syrup of fruit juices. The suggest technique can reduce the thermal effect on fruit pieces substantially and be able toretain the bioactive phytochemicals in comparison to conventional canning process. However future investigation and scale up studies should be conducted on mango, pineapple and other potential fruits to optimize the process control parameters for commercial operations. The research finding presented is valuable information for the optimization and retention of these bioactive phytochemicals compounds through applicable process design.

## Figures and Tables

**Table 1:** Goodness of fit test statistics ($X^{2})$, and associated significant probabilities (in parenthesis) of the fitted curves for vitamin C and trolox equivalent antioxidant capacity(TEAC) at different internal can temperatures for the different thermal treatment durations shown in Figure 5.

Compound	Internal Can Temperature (°C)			
	111	115	119	124
Vitamin C	7.87(0.55)	12.22(0.20)	4.01(0.91)	5.43(0.79)
TEAC	6.03(0.74)	8.60(07)	5.77(0.76)	19.20(0.02)

**Table 2:** Kinetic parameters for the degradation of compounds in pineapple and mango during canning (first-order model). Values are given with their 95% confidence intervals.

Constituent	Pineapple		Mango	
	*k*_d,111_ (10^−2^) min^−1^	*E*_a_ kJ mol^−1^	*k*_d,111_ (10^−2^) min^−1^	*E*_a_ kJ mol^−1^
Vitamin C	2.4 ± 0.1	81 ± 6	2.4 ± 0.1	109 ± 6
β-carotene	1.3 ± 0.1	92 ± 5	0.9 ± 0.0	148 ± 4
Catechin	3.5 ± 0.1	35 ± 3	1.4 ± 0.1	62 ± 4
Tannic acid	3.5 ± 0.1	44 ± 2	1.0 ± 0.0	83 ± 4
Chlorogenic acid	2.7 ± 0.1	31 ± 3	1.2 ± 0.0	96 ± 4
Epicatechin	2.1 ± 0.0	25 ± 2	0.9 ± 0.0	82 ± 3
Gallic acid	2.5 ± 0.1	73 ± 3	1.4 ± 0.0	51 ± 4
TEAC	2.2 ± 0.1	106 ± 8	1.5 ± 0.1	61 ± 5
